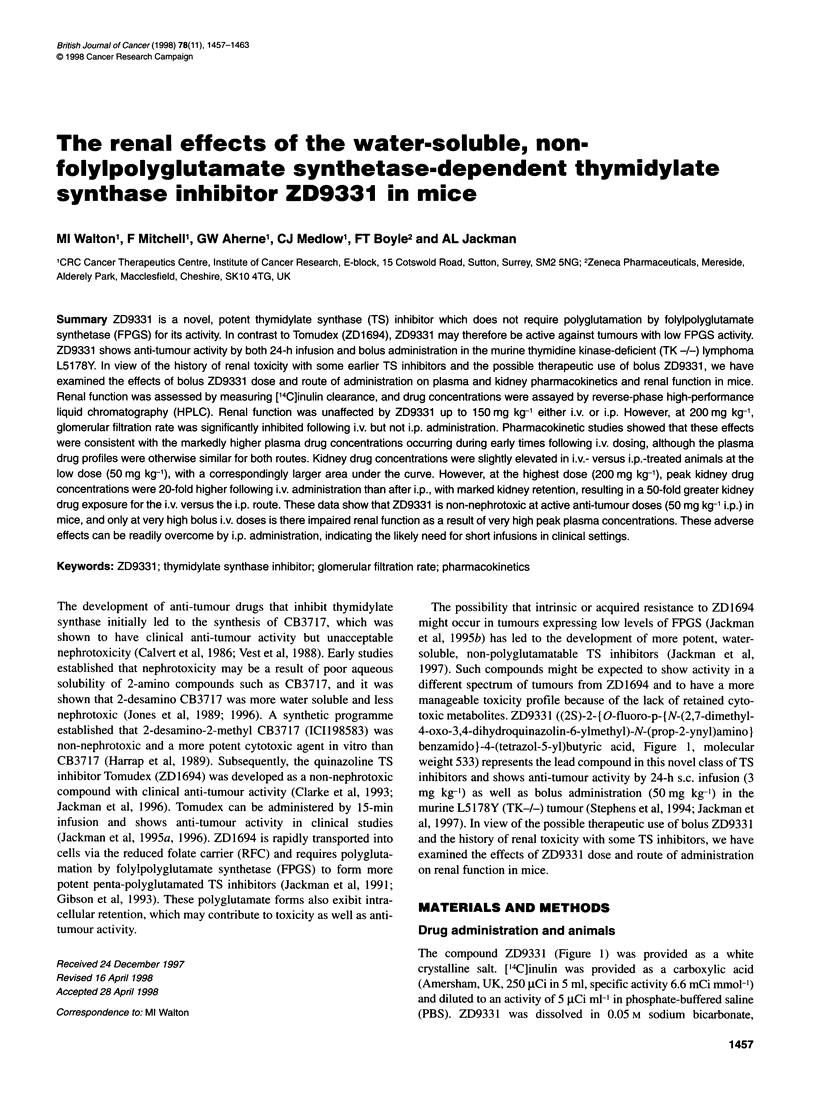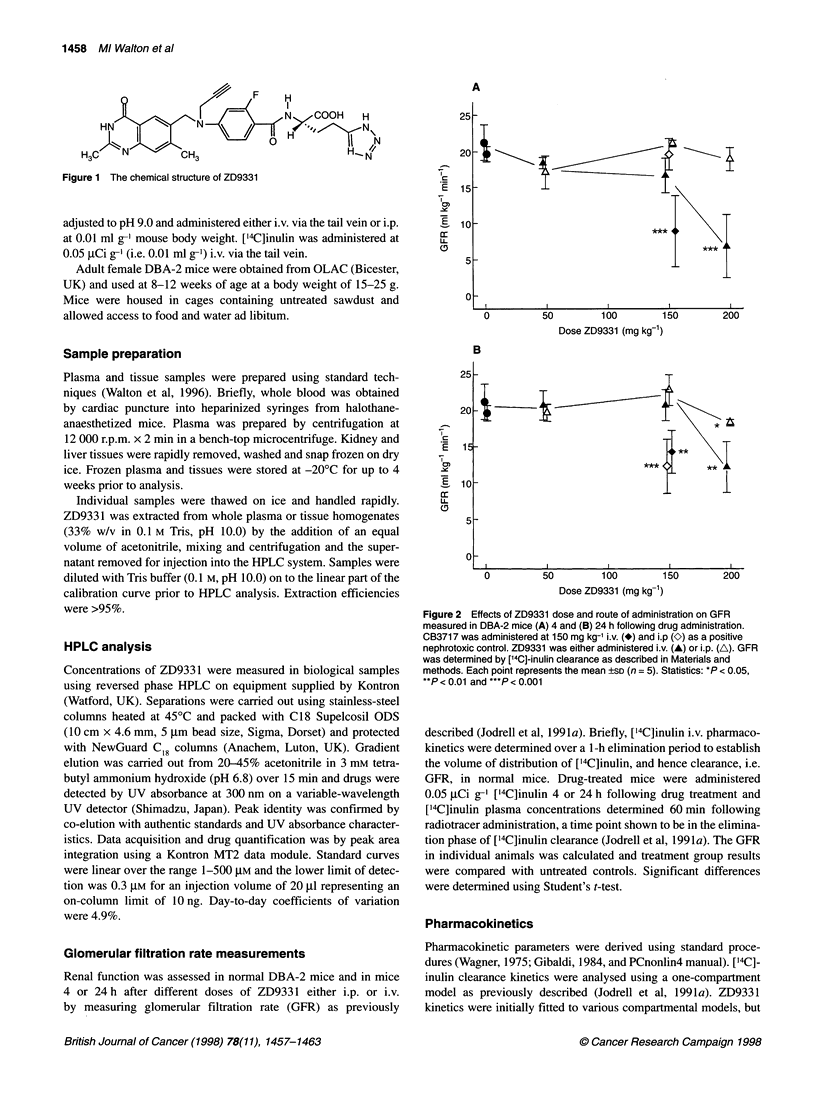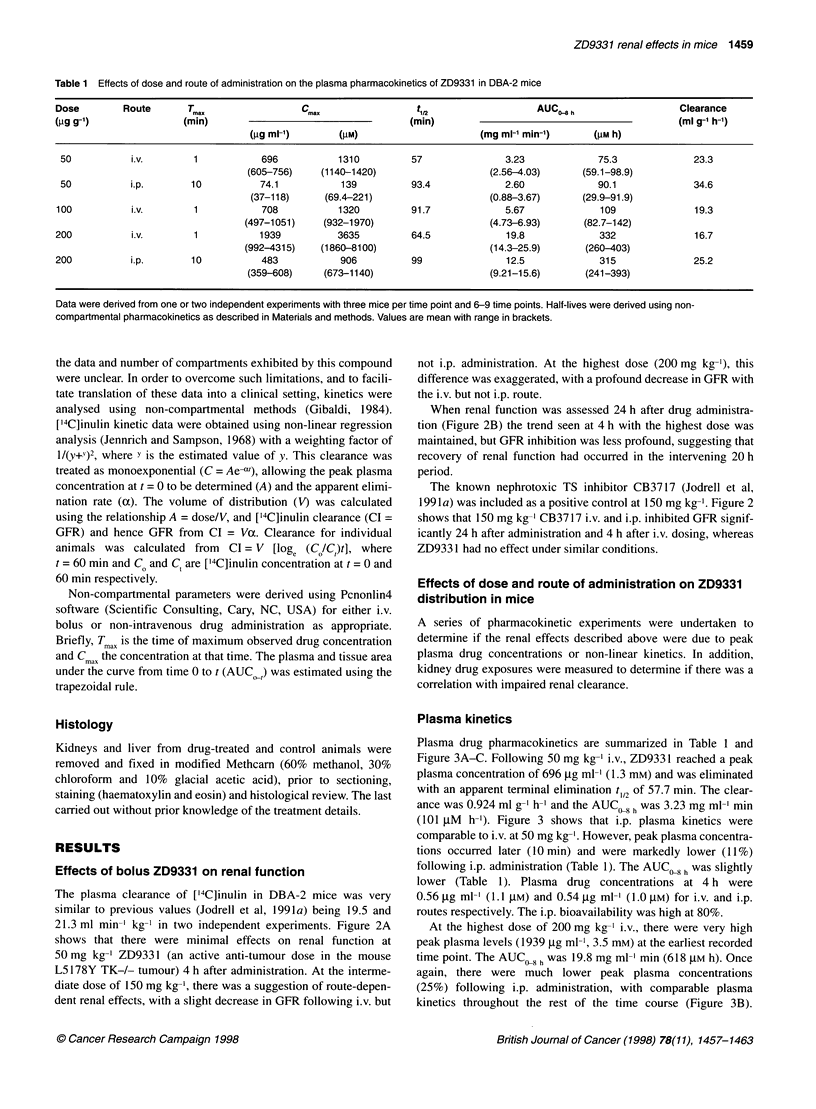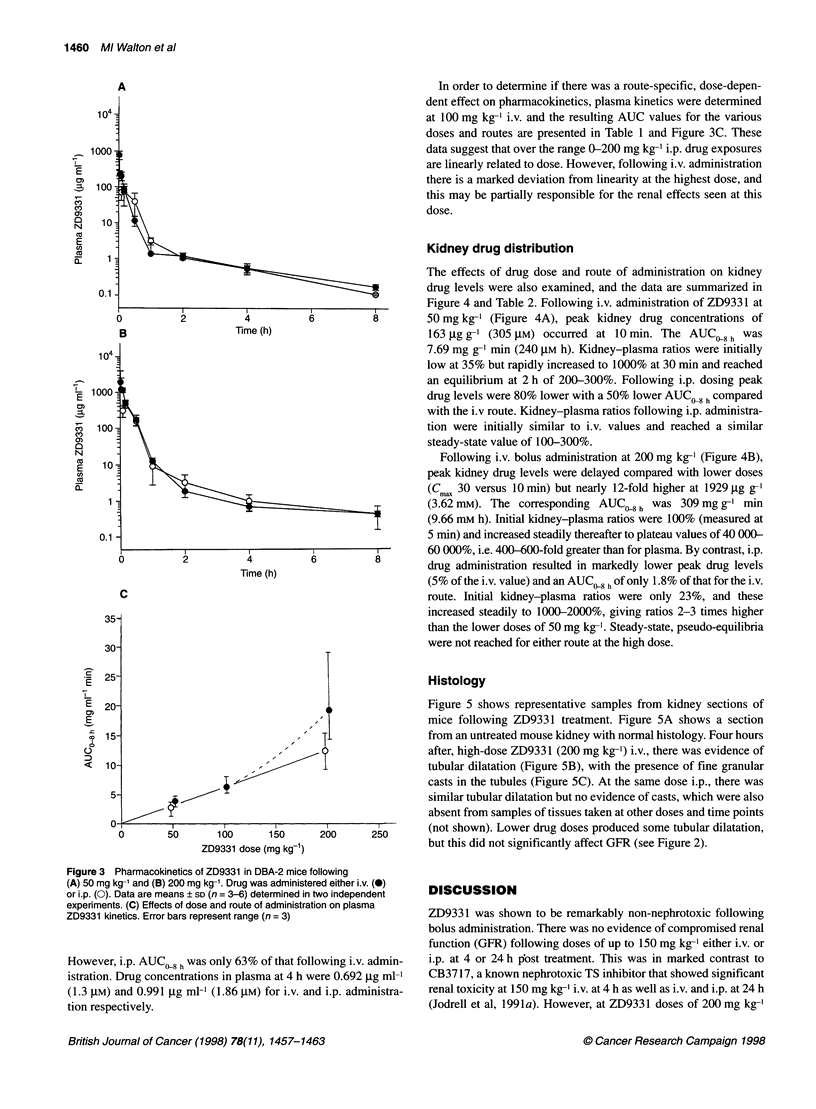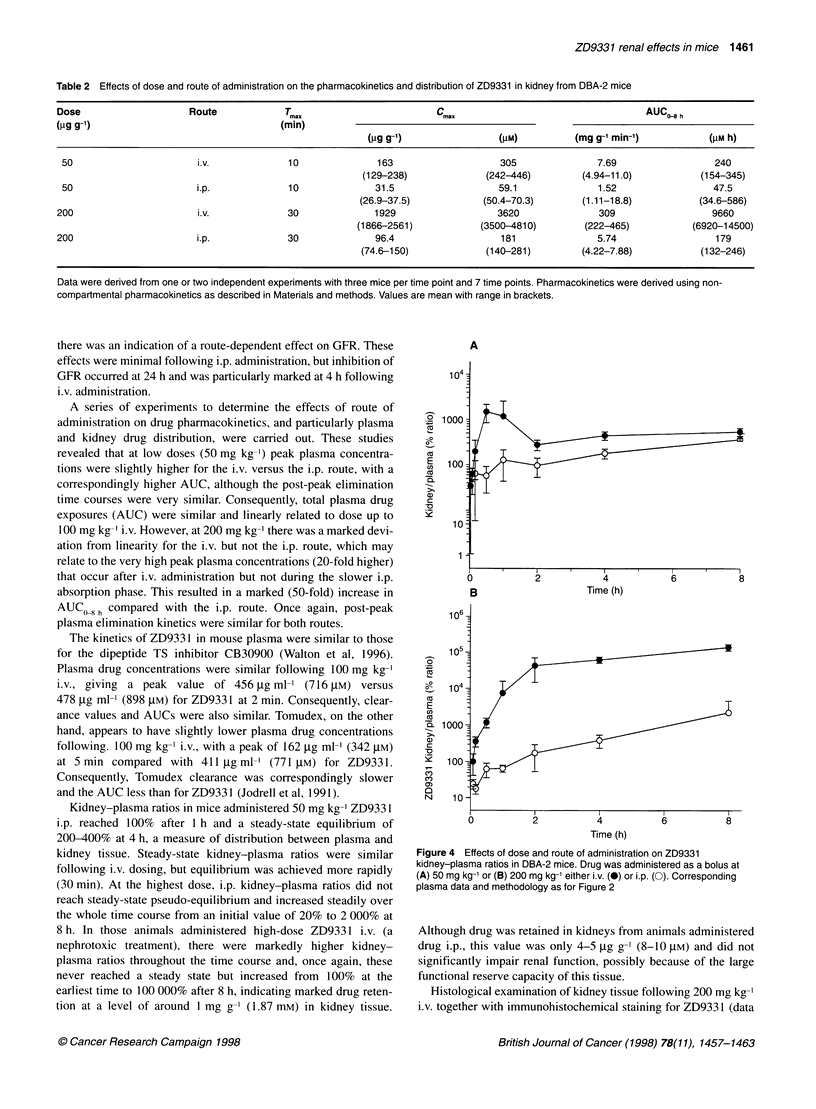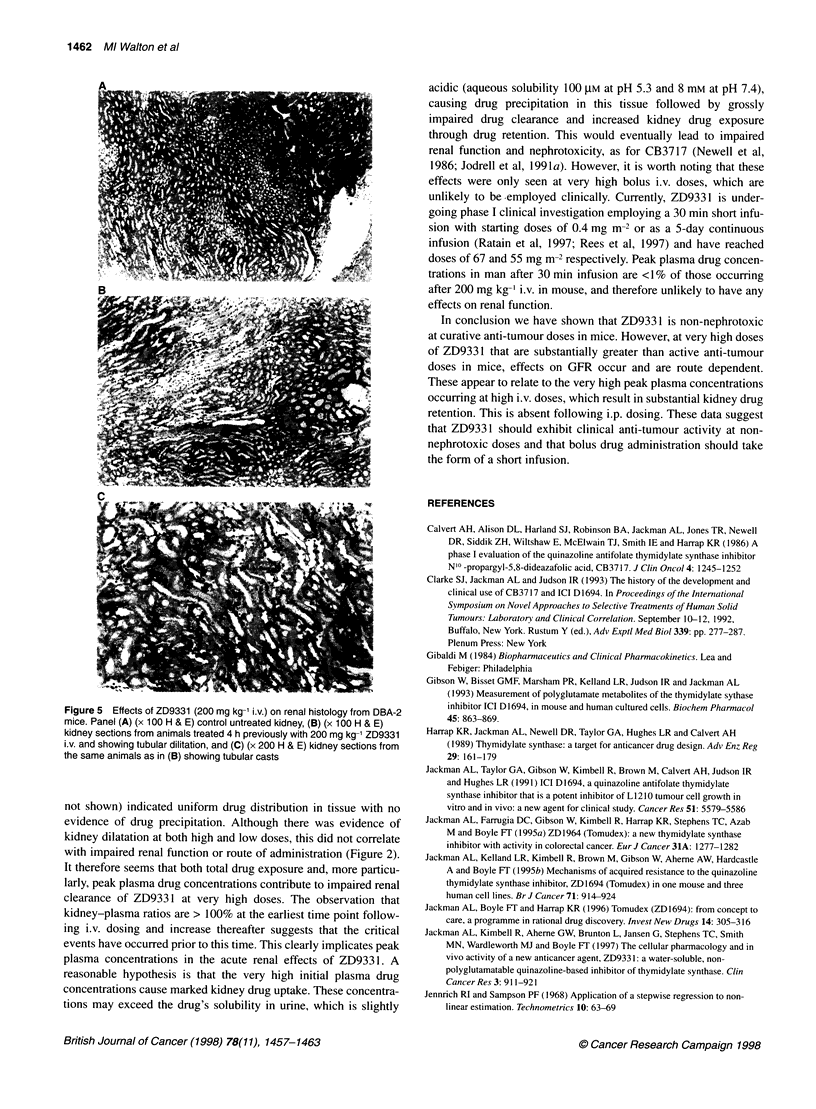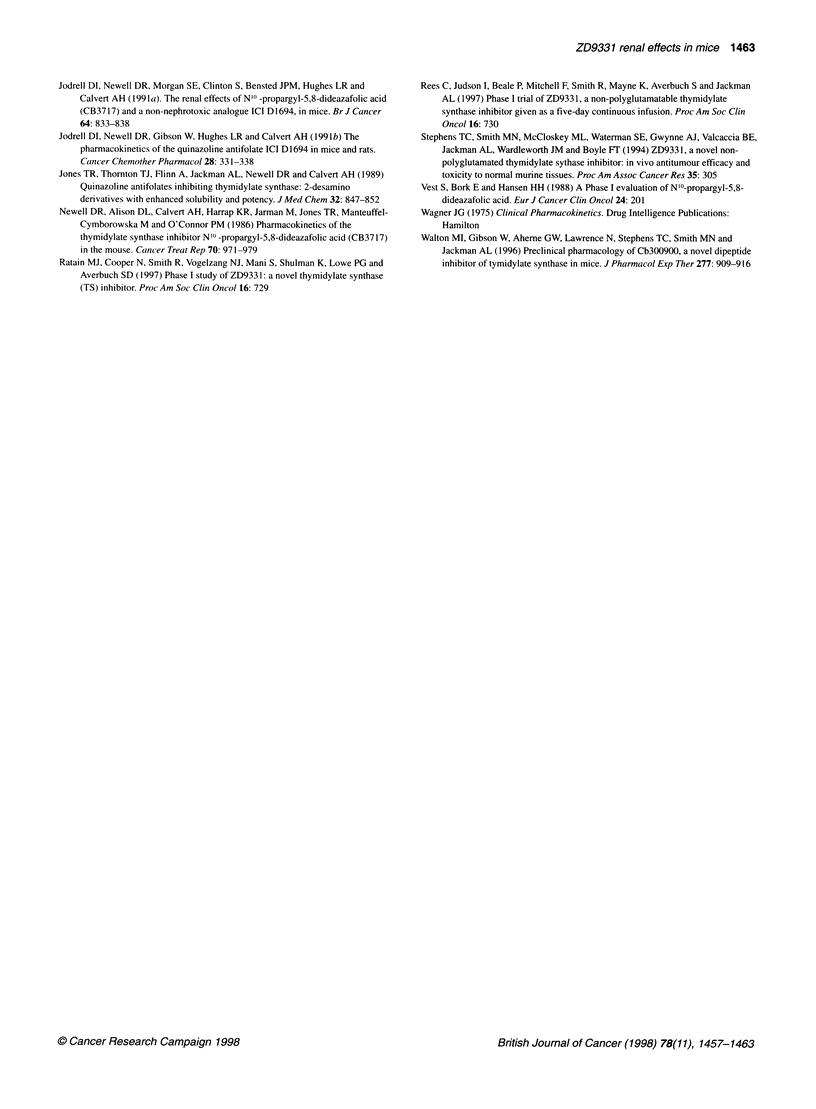# The renal effects of the water-soluble, non-folylpolyglutamate synthetase-dependent thymidylate synthase inhibitor ZD9331 in mice.

**DOI:** 10.1038/bjc.1998.707

**Published:** 1998-12

**Authors:** M. I. Walton, F. Mitchell, G. W. Aherne, C. J. Medlow, F. T. Boyle, A. L. Jackman

**Affiliations:** CRC Cancer Therapeutics Centre, Institute of Cancer Research, Sutton, Surrey, UK.

## Abstract

**Images:**


British Journal of Cancer (1998) 78(11), 1457–1463
© 1998 Cancer Research Campaign

### The renal effects of the water-soluble, non-folylpolyglutamate synthetase-dependent thymidylate synthase inhibitor ZD9331 in mice

MI Walton[1], F Mitchell[1], GW Aherne[1], CJ Medlow[1], FT Boyle[2] and AL Jackman

[1]CRC Cancer Therapeutics Centre, Institute of Cancer Research, E-block, 15 Cotswold Road, Sutton, Surrey, SM2 5NG; [2]Zeneca Pharmaceuticals, Mereside, Alderely Park, Macclesfield, Cheshire, SK10 4TG, UK

**Summary** ZD9331 is a novel, potent thymidylate synthase (TS) inhibitor which does not require polyglutamation by folylpolyglutamate synthetase (FPGS) for its activity. In contrast to Tomudex (ZD1694), ZD9331 may therefore be active against tumours with low FPGS activity. ZD9331 shows anti-tumour activity by both 24-h infusion and bolus administration in the murine thymidine kinase-deficient (TK –/–) lymphoma L5178Y. In view of the history of renal toxicity with some earlier TS inhibitors and the possible therapeutic use of bolus ZD9331, we have examined the effects of bolus ZD9331 dose and route of administration on plasma and kidney pharmacokinetics and renal function in mice. Renal function was assessed by measuring [$^{14}$C]inulin clearance, and drug concentrations were assayed by reverse-phase high-performance liquid chromatography (HPLC). Renal function was unaffected by ZD9331 up to 150 mg kg$^{-1}$ either i.v. or i.p. However, at 200 mg kg$^{-1}$, glomerular filtration rate was significantly inhibited following i.v. but not i.p. administration. Pharmacokinetic studies showed that these effects were consistent with the markedly higher plasma drug concentrations occurring during early times following i.v. dosing, although the plasma drug profiles were otherwise similar for both routes. Kidney drug concentrations were slightly elevated in i.v.- versus i.p.-treated animals at the low dose (50 mg kg$^{-1}$), with a correspondingly larger area under the curve. However, at the highest dose (200 mg kg$^{-1}$), peak kidney drug concentrations were 20-fold higher following i.v. administration than after i.p., with marked kidney retention, resulting in a 50-fold greater kidney drug exposure for the i.v. versus the i.p. route. These data show that ZD9331 is non-nephrotoxic at active anti-tumour doses (50 mg kg$^{-1}$ i.p.) in mice, and only at very high bolus i.v. doses is there impaired renal function as a result of very high peak plasma concentrations. These adverse effects can be readily overcome by i.p. administration, indicating the likely need for short infusions in clinical settings.

**Keywords:** ZD9331; thymidylate synthase inhibitor; glomerular filtration rate; pharmacokinetics

The development of anti-tumour drugs that inhibit thymidylate synthase initially led to the synthesis of CB3717, which was shown to have clinical anti-tumour activity but unacceptable nephrotoxicity (Calvert et al, 1986; Vest et al, 1988). Early studies established that nephrotoxicity may be a result of poor aqueous solubility of 2-amino compounds such as CB3717, and it was shown that 2-desamino CB3717 was more water soluble and less nephrotoxic (Jones et al, 1989; 1996). A synthetic programme established that 2-desamino-2-methyl CB3717 (ICI198583) was non-nephrotoxic and a more potent cytotoxic agent in vitro than CB3717 (Harrap et al, 1989). Subsequently, the quinazoline TS inhibitor Tomudex (ZD1694) was developed as a non-nephrotoxic compound with clinical anti-tumour activity (Clarke et al, 1993; Jackman et al, 1996). Tomudex can be administered by 15-min infusion and shows anti-tumour activity in clinical studies (Jackman et al, 1995*a*, 1996). ZD1694 is rapidly transported into cells via the reduced folate carrier (RFC) and requires polyglutamation by folylpolyglutamate synthetase (FPGS) to form more potent penta-polyglutamated TS inhibitors (Jackman et al, 1991; Gibson et al, 1993). These polyglutamate forms also exibit intracellular retention, which may contribute to toxicity as well as anti-tumour activity.

*Received 24 December 1997*
*Revised 16 April 1998*
*Accepted 28 April 1998*

*Correspondence to:* MI Walton

The possibility that intrinsic or acquired resistance to ZD1694 might occur in tumours expressing low levels of FPGS (Jackman et al, 1995*b*) has led to the development of more potent, water-soluble, non-polyglutamatable TS inhibitors (Jackman et al, 1997). Such compounds might be expected to show activity in a different spectrum of tumours from ZD1694 and to have a more manageable toxicity profile because of the lack of retained cytotoxic metabolites. ZD9331 ((2S)-2-{*O*-fluoro-p-{*N*-(2,7-dimethyl-4-oxo-3,4-dihydroquinazolin-6-ylmethyl)-*N*-(prop-2-ynyl)amino} benzamido}-4-(tetrazol-5-yl)butyric acid, Figure 1, molecular weight 533) represents the lead compound in this novel class of TS inhibitors and shows anti-tumour activity by 24-h s.c. infusion (3 mg kg$^{-1}$) as well as bolus administration (50 mg kg$^{-1}$) in the murine L5178Y (TK–/–) tumour (Stephens et al, 1994; Jackman et al, 1997). In view of the possible therapeutic use of bolus ZD9331 and the history of renal toxicity with some TS inhibitors, we have examined the effects of ZD9331 dose and route of administration on renal function in mice.

### MATERIALS AND METHODS

### Drug administration and animals

The compound ZD9331 (Figure 1) was provided as a white crystalline salt. [$^{14}$C]inulin was provided as a carboxylic acid (Amersham, UK, 250 μCi in 5 ml, specific activity 6.6 mCi mmol$^{-1}$) and diluted to an activity of 5 μCi ml$^{-1}$ in phosphate-buffered saline (PBS). ZD9331 was dissolved in 0.05 M sodium bicarbonate,

**Figure 1** The chemical structure of ZD9331

adjusted to pH 9.0 and administered either i.v. via the tail vein or i.p. at 0.01 ml $g^{-1}$ mouse body weight. [$^{14}$C]inulin was administered at 0.05 µCi $g^{-1}$ (i.e. 0.01 ml $g^{-1}$) i.v. via the tail vein.

Adult female DBA-2 mice were obtained from OLAC (Bicester, UK) and used at 8–12 weeks of age at a body weight of 15–25 g. Mice were housed in cages containing untreated sawdust and allowed access to food and water ad libitum.

### Sample preparation

Plasma and tissue samples were prepared using standard techniques (Walton et al, 1996). Briefly, whole blood was obtained by cardiac puncture into heparinized syringes from halothane-anaesthetized mice. Plasma was prepared by centrifugation at 12 000 r.p.m. × 2 min in a bench-top microcentrifuge. Kidney and liver tissues were rapidly removed, washed and snap frozen on dry ice. Frozen plasma and tissues were stored at –20°C for up to 4 weeks prior to analysis.

Individual samples were thawed on ice and handled rapidly. ZD9331 was extracted from whole plasma or tissue homogenates (33% w/v in 0.1 M Tris, pH 10.0) by the addition of an equal volume of acetonitrile, mixing and centrifugation and the supernatant removed for injection into the HPLC system. Samples were diluted with Tris buffer (0.1 M, pH 10.0) on to the linear part of the calibration curve prior to HPLC analysis. Extraction efficiencies were >95%.

### HPLC analysis

Concentrations of ZD9331 were measured in biological samples using reversed phase HPLC on equipment supplied by Kontron (Watford, UK). Separations were carried out using stainless-steel columns heated at 45°C and packed with C18 Supelcosil ODS (10 cm × 4.6 mm, 5 µm bead size, Sigma, Dorset) and protected with NewGuard $C_{18}$ columns (Anachem, Luton, UK). Gradient elution was carried out from 20–45% acetonitrile in 3 mM tetrabutyl ammonium hydroxide (pH 6.8) over 15 min and drugs were detected by UV absorbance at 300 nm on a variable-wavelength UV detector (Shimadzu, Japan). Peak identity was confirmed by co-elution with authentic standards and UV absorbance characteristics. Data acquisition and drug quantification was by peak area integration using a Kontron MT2 data module. Standard curves were linear over the range 1–500 µM and the lower limit of detection was 0.3 µM for an injection volume of 20 µl representing an on-column limit of 10 ng. Day-to-day coefficients of variation were 4.9%.

### Glomerular filtration rate measurements

Renal function was assessed in normal DBA-2 mice and in mice 4 or 24 h after different doses of ZD9331 either i.p. or i.v. by measuring glomerular filtration rate (GFR) as previously described (Jodrell et al, 1991*a*). Briefly, [$^{14}$C]inulin i.v. pharmacokinetics were determined over a 1-h elimination period to establish the volume of distribution of [$^{14}$C]inulin, and hence clearance, i.e. GFR, in normal mice. Drug-treated mice were administered 0.05 µCi $g^{-1}$ [$^{14}$C]inulin 4 or 24 h following drug treatment and [$^{14}$C]inulin plasma concentrations determined 60 min following radiotracer administration, a time point shown to be in the elimination phase of [$^{14}$C]inulin clearance (Jodrell et al, 1991*a*). The GFR in individual animals was calculated and treatment group results were compared with untreated controls. Significant differences were determined using Student's *t*-test.

**Figure 2** Effects of ZD9331 dose and route of administration on GFR measured in DBA-2 mice (**A**) 4 and (**B**) 24 h following drug administration. CB3717 was administered at 150 mg $kg^{-1}$ i.v. (◆) and i.p (◇) as a positive nephrotoxic control. ZD9331 was either administered i.v. (▲) or i.p. (△). GFR was determined by [$^{14}$C]-inulin clearance as described in Materials and methods. Each point represents the mean ±SD ($n$ = 5). Statistics: $^{*}P < 0.05$, $^{**}P < 0.01$ and $^{***}P < 0.001$

### Pharmacokinetics

Pharmacokinetic parameters were derived using standard procedures (Wagner, 1975; Gibaldi, 1984, and PCnonlin4 manual). [$^{14}$C]-inulin clearance kinetics were analysed using a one-compartment model as previously described (Jodrell et al, 1991*a*). ZD9331 kinetics were initially fitted to various compartmental models, but

© Cancer Research Campaign 1998

**Table 1** Effects of dose and route of administration on the plasma pharmacokinetics of ZD9331 in DBA-2 mice

| Dose ($\mu$g g$^{-1}$) | Route | $T_{max}$ (min) | $C_{max}$ ($\mu$g ml$^{-1}$) | $C_{max}$ ($\mu$M) | $t_{1/2}$ (min) | $AUC_{0-8\ h}$ (mg ml$^{-1}$ min$^{-1}$) | $AUC_{0-8\ h}$ ($\mu$M h) | Clearance (ml g$^{-1}$ h$^{-1}$) |
|---|---|---|---|---|---|---|---|---|
| 50 | i.v. | 1 | 696 (605–756) | 1310 (1140–1420) | 57 | 3.23 (2.56–4.03) | 75.3 (59.1–98.9) | 23.3 |
| 50 | i.p. | 10 | 74.1 (37–118) | 139 (69.4–221) | 93.4 | 2.60 (0.88–3.67) | 90.1 (29.9–91.9) | 34.6 |
| 100 | i.v. | 1 | 708 (497–1051) | 1320 (932–1970) | 91.7 | 5.67 (4.73–6.93) | 109 (82.7–142) | 19.3 |
| 200 | i.v. | 1 | 1939 (992–4315) | 3635 (1860–8100) | 64.5 | 19.8 (14.3–25.9) | 332 (260–403) | 16.7 |
| 200 | i.p. | 10 | 483 (359–608) | 906 (673–1140) | 99 | 12.5 (9.21–15.6) | 315 (241–393) | 25.2 |

Data were derived from one or two independent experiments with three mice per time point and 6–9 time points. Half-lives were derived using non-compartmental pharmacokinetics as described in Materials and methods. Values are mean with range in brackets.

the data and number of compartments exhibited by this compound were unclear. In order to overcome such limitations, and to facilitate translation of these data into a clinical setting, kinetics were analysed using non-compartmental methods (Gibaldi, 1984). [$^{14}$C]inulin kinetic data were obtained using non-linear regression analysis (Jennrich and Sampson, 1968) with a weighting factor of $1/(y+^{y})^2$, where $^{y}$ is the estimated value of $y$. This clearance was treated as monoexponential ($C = Ae^{-\alpha t}$), allowing the peak plasma concentration at $t = 0$ to be determined ($A$) and the apparent elimination rate ($\alpha$). The volume of distribution ($V$) was calculated using the relationship $A$ = dose/$V$, and [$^{14}$C]inulin clearance (CI = GFR) and hence GFR from CI = $V\alpha$. Clearance for individual animals was calculated from CI = $V$ [$\log_e$ $(C_0/C_t)t$], where $t$ = 60 min and $C_0$ and $C_t$ are [$^{14}$C]inulin concentration at $t$ = 0 and 60 min respectively.

Non-compartmental parameters were derived using Pcnonlin4 software (Scientific Consulting, Cary, NC, USA) for either i.v. bolus or non-intravenous drug administration as appropriate. Briefly, $T_{max}$ is the time of maximum observed drug concentration and $C_{max}$ the concentration at that time. The plasma and tissue area under the curve from time 0 to $t$ ($AUC_{0-t}$) was estimated using the trapezoidal rule.

### Histology

Kidneys and liver from drug-treated and control animals were removed and fixed in modified Methcarn (60% methanol, 30% chloroform and 10% glacial acetic acid), prior to sectioning, staining (haematoxylin and eosin) and histological review. The last carried out without prior knowledge of the treatment details.

### RESULTS

### Effects of bolus ZD9331 on renal function

The plasma clearance of [$^{14}$C]inulin in DBA-2 mice was very similar to previous values (Jodrell et al, 1991*a*) being 19.5 and 21.3 ml min$^{-1}$ kg$^{-1}$ in two independent experiments. Figure 2A shows that there were minimal effects on renal function at 50 mg kg$^{-1}$ ZD9331 (an active anti-tumour dose in the mouse L5178Y TK–/– tumour) 4 h after administration. At the intermediate dose of 150 mg kg$^{-1}$, there was a suggestion of route-dependent renal effects, with a slight decrease in GFR following i.v. but not i.p. administration. At the highest dose (200 mg kg$^{-1}$), this difference was exaggerated, with a profound decrease in GFR with the i.v. but not i.p. route.

When renal function was assessed 24 h after drug administration (Figure 2B) the trend seen at 4 h with the highest dose was maintained, but GFR inhibition was less profound, suggesting that recovery of renal function had occurred in the intervening 20 h period.

The known nephrotoxic TS inhibitor CB3717 (Jodrell et al, 1991*a*) was included as a positive control at 150 mg kg$^{-1}$. Figure 2 shows that 150 mg kg$^{-1}$ CB3717 i.v. and i.p. inhibited GFR significantly 24 h after administration and 4 h after i.v. dosing, whereas ZD9331 had no effect under similar conditions.

### Effects of dose and route of administration on ZD9331 distribution in mice

A series of pharmacokinetic experiments were undertaken to determine if the renal effects described above were due to peak plasma drug concentrations or non-linear kinetics. In addition, kidney drug exposures were measured to determine if there was a correlation with impaired renal clearance.

### Plasma kinetics

Plasma drug pharmacokinetics are summarized in Table 1 and Figure 3A–C. Following 50 mg kg$^{-1}$ i.v., ZD9331 reached a peak plasma concentration of 696 $\mu$g ml$^{-1}$ (1.3 mM) and was eliminated with an apparent terminal elimination $t_{1/2}$ of 57.7 min. The clearance was 0.924 ml g$^{-1}$ h$^{-1}$ and the $AUC_{0-8\ h}$ was 3.23 mg ml$^{-1}$ min (101 $\mu$M h$^{-1}$). Figure 3 shows that i.p. plasma kinetics were comparable to i.v. at 50 mg kg$^{-1}$. However, peak plasma concentrations occurred later (10 min) and were markedly lower (11%) following i.p. administration (Table 1). The $AUC_{0-8\ h}$ was slightly lower (Table 1). Plasma drug concentrations at 4 h were 0.56 $\mu$g ml$^{-1}$ (1.1 $\mu$M) and 0.54 $\mu$g ml$^{-1}$ (1.0 $\mu$M) for i.v. and i.p. routes respectively. The i.p. bioavailability was high at 80%.

At the highest dose of 200 mg kg$^{-1}$ i.v., there were very high peak plasma levels (1939 $\mu$g ml$^{-1}$, 3.5 mM) at the earliest recorded time point. The $AUC_{0-8\ h}$ was 19.8 mg ml$^{-1}$ min (618 $\mu$M h). Once again, there were much lower peak plasma concentrations (25%) following i.p. administration, with comparable plasma kinetics throughout the rest of the time course (Figure 3B).

© Cancer Research Campaign 1998

**Figure 3** Pharmacokinetics of ZD9331 in DBA-2 mice following (**A**) 50 mg $kg^{-1}$ and (**B**) 200 mg $kg^{-1}$. Drug was administered either i.v. (●) or i.p. (○). Data are means ± SD ($n$ = 3–6) determined in two independent experiments. (**C**) Effects of dose and route of administration on plasma ZD9331 kinetics. Error bars represent range ($n$ = 3)

However, i.p. $AUC_{0-8\ h}$ was only 63% of that following i.v. administration. Drug concentrations in plasma at 4 h were 0.692 μg $ml^{-1}$ (1.3 μM) and 0.991 μg $ml^{-1}$ (1.86 μM) for i.v. and i.p. administration respectively.

In order to determine if there was a route-specific, dose-dependent effect on pharmacokinetics, plasma kinetics were determined at 100 mg $kg^{-1}$ i.v. and the resulting AUC values for the various doses and routes are presented in Table 1 and Figure 3C. These data suggest that over the range 0–200 mg $kg^{-1}$ i.p. drug exposures are linearly related to dose. However, following i.v. administration there is a marked deviation from linearity at the highest dose, and this may be partially responsible for the renal effects seen at this dose.

### Kidney drug distribution

The effects of drug dose and route of administration on kidney drug levels were also examined, and the data are summarized in Figure 4 and Table 2. Following i.v. administration of ZD9331 at 50 mg $kg^{-1}$ (Figure 4A), peak kidney drug concentrations of 163 μg $g^{-1}$ (305 μM) occurred at 10 min. The $AUC_{0-8\ h}$ was 7.69 mg $g^{-1}$ min (240 μM h). Kidney–plasma ratios were initially low at 35% but rapidly increased to 1000% at 30 min and reached an equilibrium at 2 h of 200–300%. Following i.p. dosing peak drug levels were 80% lower with a 50% lower $AUC_{0-8\ h}$ compared with the i.v route. Kidney–plasma ratios following i.p. administration were initially similar to i.v. values and reached a similar steady-state value of 100–300%.

Following i.v. bolus administration at 200 mg $kg^{-1}$ (Figure 4B), peak kidney drug levels were delayed compared with lower doses ($C_{max}$ 30 versus 10 min) but nearly 12-fold higher at 1929 μg $g^{-1}$ (3.62 mM). The corresponding $AUC_{0-8\ h}$ was 309 mg $g^{-1}$ min (9.66 mM h). Initial kidney–plasma ratios were 100% (measured at 5 min) and increased steadily thereafter to plateau values of 40 000–60 000%, i.e. 400–600-fold greater than for plasma. By contrast, i.p. drug administration resulted in markedly lower peak drug levels (5% of the i.v. value) and an $AUC_{0-8\ h}$ of only 1.8% of that for the i.v. route. Initial kidney–plasma ratios were only 23%, and these increased steadily to 1000–2000%, giving ratios 2–3 times higher than the lower doses of 50 mg $kg^{-1}$. Steady-state, pseudo-equilibria were not reached for either route at the high dose.

### Histology

Figure 5 shows representative samples from kidney sections of mice following ZD9331 treatment. Figure 5A shows a section from an untreated mouse kidney with normal histology. Four hours after, high-dose ZD9331 (200 mg $kg^{-1}$) i.v., there was evidence of tubular dilatation (Figure 5B), with the presence of fine granular casts in the tubules (Figure 5C). At the same dose i.p., there was similar tubular dilatation but no evidence of casts, which were also absent from samples of tissues taken at other doses and time points (not shown). Lower drug doses produced some tubular dilatation, but this did not significantly affect GFR (see Figure 2).

### DISCUSSION

ZD9331 was shown to be remarkably non-nephrotoxic following bolus administration. There was no evidence of compromised renal function (GFR) following doses of up to 150 mg $kg^{-1}$ either i.v. or i.p. at 4 or 24 h post treatment. This was in marked contrast to CB3717, a known nephrotoxic TS inhibitor that showed significant renal toxicity at 150 mg $kg^{-1}$ i.v. at 4 h as well as i.v. and i.p. at 24 h (Jodrell et al, 1991*a*). However, at ZD9331 doses of 200 mg $kg^{-1}$

© Cancer Research Campaign 1998

**Table 2** Effects of dose and route of administration on the pharmacokinetics and distribution of ZD9331 in kidney from DBA-2 mice

| Dose (μg g⁻¹) | Route | $T_{max}$ (min) | $C_{max}$ | | $AUC_{0-8\ h}$ | |
|---|---|---|---|---|---|---|
| | | | (μg g⁻¹) | (μM) | (mg g⁻¹ min⁻¹) | (μM h) |
| 50 | i.v. | 10 | 163 (129–238) | 305 (242–446) | 7.69 (4.94–11.0) | 240 (154–345) |
| 50 | i.p. | 10 | 31.5 (26.9–37.5) | 59.1 (50.4–70.3) | 1.52 (1.11–18.8) | 47.5 (34.6–586) |
| 200 | i.v. | 30 | 1929 (1866–2561) | 3620 (3500–4810) | 309 (222–465) | 9660 (6920–14500) |
| 200 | i.p. | 30 | 96.4 (74.6–150) | 181 (140–281) | 5.74 (4.22–7.88) | 179 (132–246) |

Data were derived from one or two independent experiments with three mice per time point and 7 time points. Pharmacokinetics were derived using non-compartmental pharmacokinetics as described in Materials and methods. Values are mean with range in brackets.

there was an indication of a route-dependent effect on GFR. These effects were minimal following i.p. administration, but inhibition of GFR occurred at 24 h and was particularly marked at 4 h following i.v. administration.

A series of experiments to determine the effects of route of administration on drug pharmacokinetics, and particularly plasma and kidney drug distribution, were carried out. These studies revealed that at low doses (50 mg kg⁻¹) peak plasma concentrations were slightly higher for the i.v. versus the i.p. route, with a correspondingly higher AUC, although the post-peak elimination time courses were very similar. Consequently, total plasma drug exposures (AUC) were similar and linearly related to dose up to 100 mg kg⁻¹ i.v. However, at 200 mg kg⁻¹ there was a marked deviation from linearity for the i.v. but not the i.p. route, which may relate to the very high peak plasma concentrations (20-fold higher) that occur after i.v. administration but not during the slower i.p. absorption phase. This resulted in a marked (50-fold) increase in $AUC_{0-8\ h}$ compared with the i.p. route. Once again, post-peak plasma elimination kinetics were similar for both routes.

The kinetics of ZD9331 in mouse plasma were similar to those for the dipeptide TS inhibitor CB30900 (Walton et al, 1996). Plasma drug concentrations were similar following 100 mg kg⁻¹ i.v., giving a peak value of 456 μg ml⁻¹ (716 μM) versus 478 μg ml⁻¹ (898 μM) for ZD9331 at 2 min. Consequently, clearance values and AUCs were also similar. Tomudex, on the other hand, appears to have slightly lower plasma drug concentrations following. 100 mg kg⁻¹ i.v., with a peak of 162 μg ml⁻¹ (342 μM) at 5 min compared with 411 μg ml⁻¹ (771 μM) for ZD9331. Consequently, Tomudex clearance was correspondingly slower and the AUC less than for ZD9331 (Jodrell et al, 1991).

Kidney–plasma ratios in mice administered 50 mg kg⁻¹ ZD9331 i.p. reached 100% after 1 h and a steady-state equilibrium of 200–400% at 4 h, a measure of distribution between plasma and kidney tissue. Steady-state kidney–plasma ratios were similar following i.v. dosing, but equilibrium was achieved more rapidly (30 min). At the highest dose, i.p. kidney–plasma ratios did not reach steady-state pseudo-equilibrium and increased steadily over the whole time course from an initial value of 20% to 2 000% at 8 h. In those animals administered high-dose ZD9331 i.v. (a nephrotoxic treatment), there were markedly higher kidney–plasma ratios throughout the time course and, once again, these never reached a steady state but increased from 100% at the earliest time to 100 000% after 8 h, indicating marked drug retention at a level of around 1 mg g⁻¹ (1.87 mM) in kidney tissue.

**Figure 4** Effects of dose and route of administration on ZD9331 kidney–plasma ratios in DBA-2 mice. Drug was administered as a bolus at (**A**) 50 mg kg⁻¹ or (**B**) 200 mg kg⁻¹ either i.v. (●) or i.p. (○). Corresponding plasma data and methodology as for Figure 2

Although drug was retained in kidneys from animals administered drug i.p., this value was only 4–5 μg g⁻¹ (8–10 μM) and did not significantly impair renal function, possibly because of the large functional reserve capacity of this tissue.

Histological examination of kidney tissue following 200 mg kg⁻¹ i.v. together with immunohistochemical staining for ZD9331 (data

© Cancer Research Campaign 1998

**Figure 5** Effects of ZD9331 (200 mg $kg^{-1}$ i.v.) on renal histology from DBA-2 mice. Panel (**A**) (× 100 H & E) control untreated kidney, (**B**) (× 100 H & E) kidney sections from animals treated 4 h previously with 200 mg $kg^{-1}$ ZD9331 i.v. and showing tubular dilitation, and (**C**) (× 200 H & E) kidney sections from the same animals as in (**B**) showing tubular casts not shown) indicated uniform drug distribution in tissue with no evidence of drug precipitation. Although there was evidence of kidney dilatation at both high and low doses, this did not correlate with impaired renal function or route of administration (Figure 2). It therefore seems that both total drug exposure and, more particularly, peak plasma drug concentrations contribute to impaired renal clearance of ZD9331 at very high doses. The observation that kidney–plasma ratios are > 100% at the earliest time point following i.v. dosing and increase thereafter suggests that the critical events have occurred prior to this time. This clearly implicates peak plasma concentrations in the acute renal effects of ZD9331. A reasonable hypothesis is that the very high initial plasma drug concentrations cause marked kidney drug uptake. These concentrations may exceed the drug's solubility in urine, which is slightly acidic (aqueous solubility 100 μM at pH 5.3 and 8 mM at pH 7.4), causing drug precipitation in this tissue followed by grossly impaired drug clearance and increased kidney drug exposure through drug retention. This would eventually lead to impaired renal function and nephrotoxicity, as for CB3717 (Newell et al, 1986; Jodrell et al, 1991*a*). However, it is worth noting that these effects were only seen at very high bolus i.v. doses, which are unlikely to be employed clinically. Currently, ZD9331 is undergoing phase I clinical investigation employing a 30 min short infusion with starting doses of 0.4 mg $m^{-2}$ or as a 5-day continuous infusion (Ratain et al, 1997; Rees et al, 1997) and have reached doses of 67 and 55 mg $m^{-2}$ respectively. Peak plasma drug concentrations in man after 30 min infusion are <1% of those occurring after 200 mg $kg^{-1}$ i.v. in mouse, and therefore unlikely to have any effects on renal function.

In conclusion we have shown that ZD9331 is non-nephrotoxic at curative anti-tumour doses in mice. However, at very high doses of ZD9331 that are substantially greater than active anti-tumour doses in mice, effects on GFR occur and are route dependent. These appear to relate to the very high peak plasma concentrations occurring at high i.v. doses, which result in substantial kidney drug retention. This is absent following i.p. dosing. These data suggest that ZD9331 should exhibit clinical anti-tumour activity at non-nephrotoxic doses and that bolus drug administration should take the form of a short infusion.

### REFERENCES

Calvert AH, Alison DL, Harland SJ, Robinson BA, Jackman AL, Jones TR, Newell DR, Siddik ZH, Wiltshaw E, McElwain TJ, Smith IE and Harrap KR (1986) A phase I evaluation of the quinazoline antifolate thymidylate synthase inhibitor $N^{10}$-propargyl-5,8-dideazafolic acid, CB3717. *J Clin Oncol* **4**: 1245–1252

Clarke SJ, Jackman AL and Judson IR (1993) The history of the development and clinical use of CB3717 and ICI D1694. In *Proceedings of the International Symposium on Novel Approaches to Selective Treatments of Human Solid Tumours: Laboratory and Clinical Correlation*. September 10–12, 1992, Buffalo, New York. Rustum Y (ed.), *Adv Exptl Med Biol* **339**: pp. 277–287. Plenum Press: New York

Gibaldi M (1984) *Biopharmaceutics and Clinical Pharmacokinetics*. Lea and Febiger: Philadelphia

Gibson W, Bisset GMF, Marsham PR, Kelland LR, Judson IR and Jackman AL (1993) Measurement of polyglutamate metabolites of the thymidylate sythase inhibitor ICI D1694, in mouse and human cultured cells. *Biochem Pharmacol* **45**: 863–869.

Harrap KR, Jackman AL, Newell DR, Taylor GA, Hughes LR and Calvert AH (1989) Thymidylate synthase: a target for anticancer drug design. *Adv Enz Reg* **29**: 161–179

Jackman AL, Taylor GA, Gibson W, Kimbell R, Brown M, Calvert AH, Judson IR and Hughes LR (1991) ICI D1694, a quinazoline antifolate thymidylate synthase inhibitor that is a potent inhibitor of L1210 tumour cell growth in vitro and in vivo: a new agent for clinical study. *Cancer Res* **51**: 5579–5586

Jackman AL, Farrugia DC, Gibson W, Kimbell R, Harrap KR, Stephens TC, Azab M and Boyle FT (1995*a*) ZD1964 (Tomudex): a new thymidylate synthase inhibitor with activity in colorectal cancer. *Eur J Cancer* **31A**: 1277–1282

Jackman AL, Kelland LR, Kimbell R, Brown M, Gibson W, Aherne AW, Hardcastle A and Boyle FT (1995*b*) Mechanisms of acquired resistance to the quinazoline thymidylate synthase inhibitor, ZD1694 (Tomudex) in one mouse and three human cell lines. *Br J Cancer* **71**: 914–924

Jackman AL, Boyle FT and Harrap KR (1996) Tomudex (ZD1694): from concept to care, a programme in rational drug discovery. *Invest New Drugs* **14**: 305–316

Jackman AL, Kimbell R, Aherne GW, Brunton L, Jansen G, Stephens TC, Smith MN, Wardleworth MJ and Boyle FT (1997) The cellular pharmacology and in vivo activity of a new anticancer agent, ZD9331: a water-soluble, non-polyglutamatable quinazoline-based inhibitor of thymidylate synthase. *Clin Cancer Res* **3**: 911–921

Jennrich RI and Sampson PF (1968) Application of a stepwise regression to non-linear estimation. *Technometrics* **10**: 63–69

© Cancer Research Campaign 1998

Jodrell DI, Newell DR, Morgan SE, Clinton S, Bensted JPM, Hughes LR and Calvert AH (1991*a*). The renal effects of $N^{10}$-propargyl-5,8-dideazafolic acid (CB3717) and a non-nephrotoxic analogue ICI D1694, in mice. *Br J Cancer* **64**: 833–838

Jodrell DI, Newell DR, Gibson W, Hughes LR and Calvert AH (1991*b*) The pharmacokinetics of the quinazoline antifolate ICI D1694 in mice and rats. *Cancer Chemother Pharmacol* **28**: 331–338

Jones TR, Thornton TJ, Flinn A, Jackman AL, Newell DR and Calvert AH (1989) Quinazoline antifolates inhibiting thymidylate synthase: 2-desamino derivatives with enhanced solubility and potency. *J Med Chem* **32**: 847–852

Newell DR, Alison DL, Calvert AH, Harrap KR, Jarman M, Jones TR, Manteuffel-Cymborowska M and O'Connor PM (1986) Pharmacokinetics of the thymidylate synthase inhibitor $N^{10}$-propargyl-5,8-dideazafolic acid (CB3717) in the mouse. *Cancer Treat Rep* **70**: 971–979

Ratain MJ, Cooper N, Smith R, Vogelzang NJ, Mani S, Shulman K, Lowe PG and Averbuch SD (1997) Phase I study of ZD9331: a novel thymidylate synthase (TS) inhibitor. *Proc Am Soc Clin Oncol* **16**: 729

Rees C, Judson I, Beale P, Mitchell F, Smith R, Mayne K, Averbuch S and Jackman AL (1997) Phase I trial of ZD9331, a non-polyglutamatable thymidylate synthase inhibitor given as a five-day continuous infusion. *Proc Am Soc Clin Oncol* **16**: 730

Stephens TC, Smith MN, McCloskey ML, Waterman SE, Gwynne AJ, Valcaccia BE, Jackman AL, Wardleworth JM and Boyle FT (1994) ZD9331, a novel non-polyglutamated thymidylate sythase inhibitor: in vivo antitumour efficacy and toxicity to normal murine tissues. *Proc Am Assoc Cancer Res* **35**: 305

Vest S, Bork E and Hansen HH (1988) A Phase I evaluation of $N^{10}$-propargyl-5,8-dideazafolic acid. *Eur J Cancer Clin Oncol* **24**: 201

Wagner JG (1975) *Clinical Pharmacokinetics*. Drug Intelligence Publications: Hamilton

Walton MI, Gibson W, Aherne GW, Lawrence N, Stephens TC, Smith MN and Jackman AL (1996) Preclinical pharmacology of Cb300900, a novel dipeptide inhibitor of tymidylate synthase in mice. *J Pharmacol Exp Ther* **277**: 909–916

© Cancer Research Campaign 1998